# Effectiveness of the Fascial Manipulation Approach Associated with a Physiotherapy Program in Recurrent Shoulder Disease

**DOI:** 10.3390/life13061396

**Published:** 2023-06-15

**Authors:** Silvia Bellotti, Massimo Busato, Carla Cattaneo, Mirco Branchini

**Affiliations:** 1 Independent Researcher, 24029 Bergamo, Italy; silviabellotti@icloud.com; 2 Independent Researcher, 24060 Bergamo, Italy; mahem1971@gmail.com; 3 ASST Bergamo Est, 24068 Seriate, Italy; albycarla7173@gmail.com; 4 Independent Researcher, 40131 Bologna, Italy

**Keywords:** shoulder pain, physiotherapy program, fascial manipulation, MCID (minimal clinical important difference)

## Abstract

Shoulder pain is a serious clinical disease frequently related to absence from work. It is characterized by pain and stiffness, probably connected to the presence of an inflammatory substrate involving gleno-humeral capsule and collagen tissues. A physiotherapy program has shown to be effective for the conservative treatment of this disorder. Our aim is to assess if a manual treatment directed to fascial tissues could obtain better improvement regarding pain, strength, mobility, and function. A total of 94 healthcare workers with recurrent shoulder pain were recruited and then randomized in two groups: the control group (CG) underwent a five-session physiotherapy program; the study group (SG) underwent three sessions of physiotherapy and two sessions of fascial manipulation (FM) technique. At the end of the treatment phase, both groups improved every outcome. Despite few statistical differences between groups, at the follow-up visit, a greater percentage of subjects in SG overcame the minimal clinical important difference (MCID) in every outcome. We conclude that FM is effective for treatment of shoulder pain and further studies should better assess how to manage this treatment to obtain better results.

## 1. Introduction

Shoulder pain is an important clinical and socioeconomic problem: shoulder pain and stiffness can lead to disability that affects both the working and social spheres of the individual’s life.

A feature of this clinical condition highlighted by the literature is its persistence, in terms of duration of pain or impairments or both, and could increase in case of enlargement of the painful area, which affects the subjects for a very long time, influencing their activities for up to a year [1].

Analyzing the literature, we find that shoulder pain, similar to back pain, often affects healthcare professionals and is one of the most frequent causes of absence from work, highlighting that posture-related factors or highly repetitive activities, with flexion or abduction greater than 60 degrees, are associated with this disorder.

Moreover, a causal relationship has been found between vigorous efforts, high level of static contractions, prolonged static loads, and extreme postures, as well as combinations of these factors [2].

Other causes of shoulder pain may be related to intrinsic glenohumeral joint disease, pathology in the periarticular tissues, dysfunctions related to spine and thoracic structures, or visceral dysfunctions that cause referred pain [3].

The clinical diagnosis of “shoulder pain” is carried out through the anamnesis, considering the type and distribution of pain, and through physical examination [3].

Chronicization of this condition is common, especially when radiological investigations do not show structural lesions; it often establishes progressively, causing functional limitations, even severe in the activities of daily life [4].

The chronicity of a painful shoulder often results in a frozen shoulder, a clinical condition that has a prevalence of 2–5% in the general population.

Many studies indicate that the onset of this pathology is due to the presence of an inflammatory substrate involving the shoulder joint capsule. This is associated with increased amounts of collagen, fibrotic growth factors such as transforming growth factor beta, and inflammatory cytokines and interleukins [5].

This biological condition causes pain and consequent immobility which, in the process of becoming chronic, is further aggravated by the very fear of pain, which leads the person to further reduce their activities.

This mechanism is explained very well by the fear–avoidance model (FAM—“avoidance of movement for fear of musculoskeletal pain”), which is proposed to describe the interaction between psychological characteristics such as fear of pain and worsening of the functional activities with development of chronic musculoskeletal pain and disability [6].

Many treatments are proposed in the literature for shoulder pain; their main common goals are to restore and maintain function. Physiotherapy treatment is always present in every study. 

Consequently, to define the specific physiotherapy strategy is the primary objective to prevent the aggravation of the clinical picture.

Biomedical database research has shown good levels of evidence for a program of stretching and selective strengthening exercises to positively affect the development of shoulder pain [7,8,9]. 

Stretching exercises are intended to prevent aggravation of joint limitation and promote a faster return to normal range; selective active exercises aim at maintaining and improving the strength of the shoulder girdle.

It is possible to start this activity by proposing isometric work within the pain-free range, for example, by promoting the use of an elastic band that provides gradually increasing resistance as the range of motion increases [10].

The literature then confirmed how, in consideration of the involvement of the articular and periarticular collagenous tissues and the close continuity between these and the fascia of the muscular elements involved in this clinical condition, manual treatments aimed at restoring the best functionality of the muscular fascia can affect the recovery function of the painful shoulder [11,12].

Based on these considerations, we hypothesized a clinical trial that, in addition to these physiotherapy proposals, also includes a manual treatment aimed at the periarticular soft tissues involved in the chronicization of pain.

A manual technique that provides a rationale for treating specific areas of fascia is fascial manipulation^®^ (FM).

To choose where muscular fascia needs to be treated, FM uses a twofold analysis: at first, a movement verification (MoVe), and, second, a palpatory verification (PaVe). MoVe is carried out with provocative tests in the involved segments in all directions along the three planes (sagittal, frontal, and horizontal); PaVe is an assessment of the fascial gliding into specific fascial areas; it is carried out with manual friction on the deep fascia: positive points are characterized by resistance in manual friction: these points are usually described as densified.

In this way, the altered fascial areas are highlighted and defined by the technique as centers of coordination (CCs) and centers of fusion (CFs), which are potentially the site of dysfunction. The treatment consists of a manual deep friction intervention capable of heating the fascial tissues and fluidifying the sliding of the fascial planes of the deep fascia.

The mechanical and chemical effects of the FM manual treatment due to the deep manual friction and the subsequent local inflammatory tissue response are able to influence the extra cellular matrix (ECM) components and the thickness of the deep fascia in CC and CF areas, restoring its normal sliding and tensioning [13,14]. 

Due to the body-wide fascial tissue continuity, when stimulating a CC or a CF, we can also improve tensional adaptation in segments far from the treated areas, restoring a global tensional physiological balance.

The anatomical studies named these myofascial continuities as sequences, diagonals, or spirals. They are not only a functional concept but have an anatomical substratum of fascial continuity and muscular expansions on the fascia itself [13]. 

Considering both the clinical involvement of the connective tissues for the onset of shoulder pain and the effectiveness of FM for the treatment of different myofascial dysfunctions, we want to verify if this manual fascial treatment in association with a physiotherapy program could better improve shoulder pain in comparison with a physiotherapy program alone; this will be made up of the best evidence in the literature regarding treatments for shoulder pain [15,16,17,18].

## 2. Materials and Methods

### 2.1. Design of the Study

One of the objectives of our study is to highlight the importance of physiotherapy intervention for subjects suffering from chronic shoulder pain, through the administration of a targeted and continuous program of exercises to improve pain symptoms, ROM (range of movement), muscle recruitment, function, and quality of life in these people.

We designed a randomized, single-blind, controlled clinical trial in which the study sample was divided into two groups.

We decided to recruit the population of this study among the employees of a healthcare center in Bergamo (ASST Bergamo Est), knowing that healthcare workers’ activity is often related to recurrent pathologies of the upper limbs, and the assistance activity itself can represent a chronicization factor for pathologies affecting the scapula–humeral system [19].

The study was approved by the Provincial Ethics Committee of Bergamo, registration no. 697, dated 8 October 2016, clinicaltrials.gov identifier: NCT03041727.

The sample recruitment was carried out with an initial awareness-raising process that took place through a preliminary survey made by a questionnaire addressed to all the healthcare workers of the ASST Bergamo Est.

Interested volunteers could then contact the physiatrist in charge of evaluating the inclusion and exclusion criteria settled in the protocol for possible enrolment in research protocol [20].

We settled on these inclusion criteria: age between 30 and 60 years, shoulder pain present for at least three months with two or more points to NRS scale, and clinical positivity to at least two clinical tests among Jobe, Neer, and Hawkins.

The exclusion criteria were as follows: positive imaging (US or MRI) of a structural fracture of the glenohumeral joint, positive anamnesis of rheumatic, oncological, neurological, and psychiatric diseases, and previous fractures and/or trauma related to scapulohumeral girdle or cervical spine.

All the subjects volunteered for the study were included; after their signature of the informed consent, they were randomized at T0 into a control group (CG) and a study group (SG); the division into the two groups was carried out by an operator (PTA) through a computerized randomization system using the Microsoft Excel software [21].

T0 represents also the first assessment and the first treatment session.

Both CG and SG carried out five weekly exercise sessions (T0–T1–T2–T3–T4), and a final follow-up session (T5) one month after the fifth appointment.

**Figure A1:**
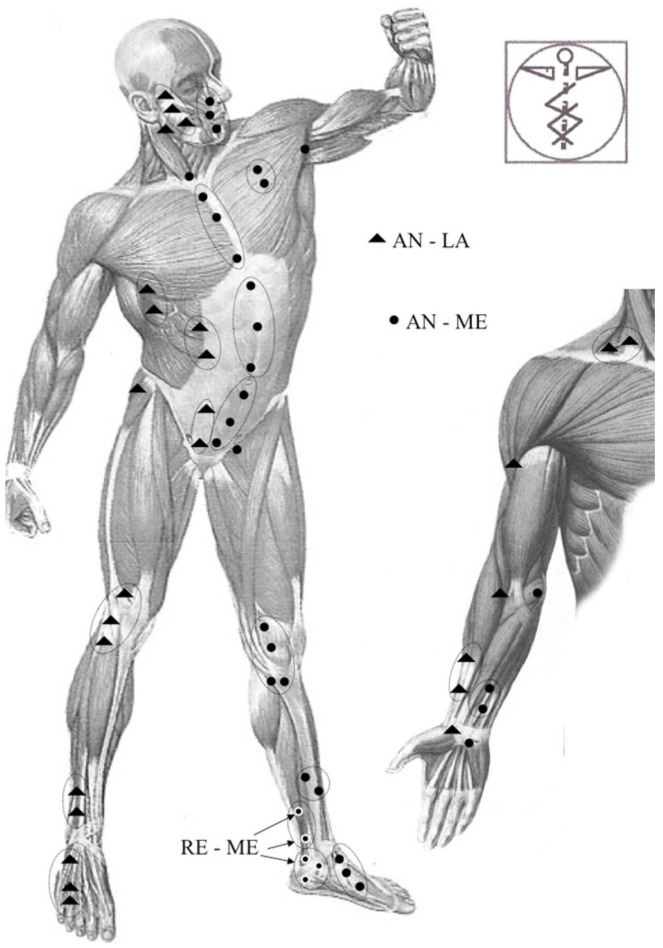
In this figure are shown CFs in the frontal aspect of the body.

**Figure A2:**
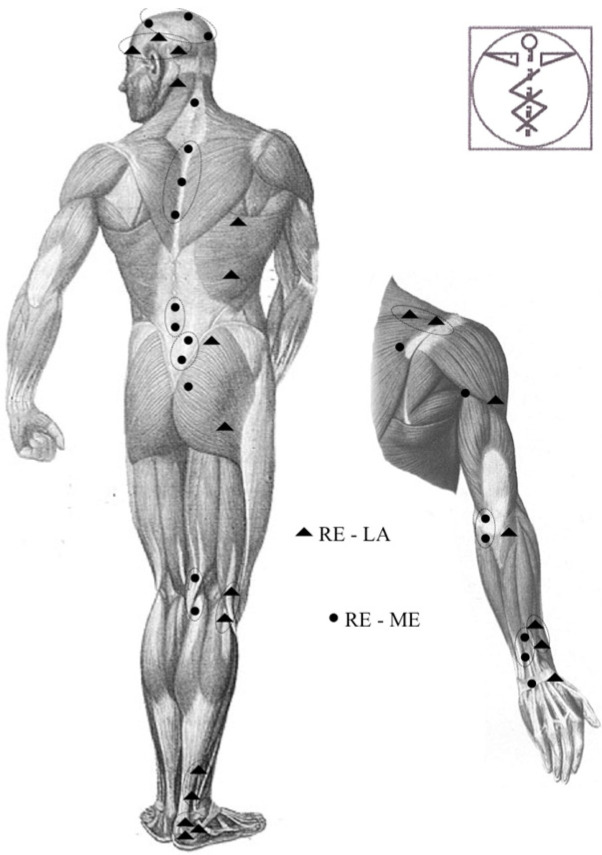
In this figure are shown CFs in the posterior aspect of the body.

SG, in the second and fifth sessions, underwent an FM treatment instead of the exercise protocol. The treatment was performed by another physiotherapist (PT C), with more than 5 years of experience in performing the FM technique. PT C treated only the CFs in their diagonal or spiral organization (as described by FM technique, excluding CCs because they are more closely related to uniplanar activities and movements [13]; see Figure A1 and Figure A2 in Appendix B).

The exercise sessions were conducted in groups of three; each group was supervised by a physiotherapist (PT B) who was experienced and trained in teaching the execution of the exercise protocol; each group involved GS or GC subjects separately. 

In each session, all subjects were recommended to perform, once a day, the exercises indicated in the first session [3], with a daily commitment of approximately 25 min [22]. 

As indicated in Appendix A, the program included six exercises, selected on the best available evidence [7]: three stretching exercises directed at upper trapezius, pectoralis minor, and posterior capsule, which were performed in three repetitions of thirty seconds each as indicated by the references [23]; and three specific strengthening exercises [24] for the shoulder external rotators, the lower trapezius, and the serratus anterior. Subjects were request to perform three series of thirty activations each in the pain-free range using an elastic band resistance; this resistance was increased in the third week and always progressively [7].

To ease the execution of this homework protocol, the exercises were supervised by PT B in each session and each participant was provided with a DVD containing the videos of the same exercises [25].

We asked each subject to keep a daily diary to stimulate adherence to the protocol exercise and to highlight any needs to adapt them [25].

Between the fifth session and the last follow-up visit, participants were asked to suspend the execution of the exercises to verify the maintenance of the functional recovery obtained over time. 

Each subject had direct live chat contact with their physiotherapist for warning of any adverse events they might have encountered during the execution of the home exercise program.

### 2.2. Primary and Secondary Outcomes

We used pain as the primary outcome of our study since it is a clinical condition potentially capable of favoring the chronicization of the clinical picture and the catastrophizing process.

We decided to standardize pain assessment using the NRS scale while performing the Appley Scratch Test [26].

Considering that a painful shoulder is usually also affected in its mobility, strength, and function, we decided to use these as secondary outcomes.

Active range of motion (aROM) was assessed using a bubble goniometer placed on the person’s wrist, measuring the following directions of movement:

- Flexion (aROM flex): individual standing in front of a square-shaped column. Shoulder flexion with elbow outstretched of the upper limb to be tested was required. The operator corrected any movement compensations (e.g., trunk movements).
- Abduction (aROM abd): individual standing upright and aligned with the same column with the side not being examined; shoulder abduction with elbow extended was required. The operator paid attention to any movement compensations.
- Internal and external rotation (aROM intra–aROM extra): subject prone on the couch, with pillow positioned under the chest and operator at the side to be tested. The humerus of the limb to be tested rested on the couch and the forearm in a vertical position outside with the elbow free to move; external rotation and internal rotation with flexed elbow were requested. The operator’s task was to check the possible movement compensation of the articular elements of the upper limb.
- Strength was measured through active muscle recruitment in flexion and abduction. The measurement was carried out by an isometric dynamometer (Sauter GmbH-FK 1K), fixed at the bottom of the wall bar by means of a fixed-length rope grasped by the hand of the limb to be tested, so as to allow a movement of less than 90° of elevation; the directions of movement considered were as follows:
- Flexion (s-Flex): The subject was positioned in an upright position facing and in contact with the back against the wall bar, requesting an elbow-stretched elevation. The operator corrected any movement compensations.
- Abduction (s-Abd): The subject was positioned with the contralateral shoulder facing the wall bar, requesting abduction elevation with the elbow outstretched, with the rope tied to the dynamometer anterior to the subject’s body. The operator checked that no movement compensations were performed.

The instruction was to make a progressive movement that places the rope in tension, expressing the maximum intensity for at least 5 s. The recorded value was deduced from the average of three successive repetitions, with a 5 s pause between them.

The operator checked that no movement compensation was performed. The instrument was previously validated through a test which involved the execution of 10 measurements with a nominal weight of 6 kg; the measurements were then performed in Newton units.

Shoulder disability and function were measured using the DASH [27] and Constant Murley [28] scales.

The initial, final, and intratreatment evaluations were always performed by the same operator, who was unaware of the attributions of the subjects in the two study groups, whereas the FM treatments were performed by a physiotherapist who did not carry out other treatments with the subjects of both groups.

The NRS, aROM, and strength outcome assessments were carried out at recruitment (T0), before (T1) and after (T2) the second session, before (T3) and after (T4) the fifth session, and at follow-up (T5) one month after the latter. Functionality and disability outcomes measured by the DASH and Constant Murley scale were measured at T0, T4, and T5. 

### 2.3. Statistical Analysis and Intention to Treat

For the statistical analysis, we calculated the sample size considering α = 0.05, β = 0.20 for the main objective of the study (pain assessed with NRS scale) and the expectation of a difference of 2 points and a SD of 3 points [24]. Due to these parameters, the sample size was calculated for 35 subjects for each arm of the study.

Data were collected and recorded in a database by the physiotherapist who assessed all subjects without knowing which group they belonged to. The database was used for a blinded analysis by another operator using STATA 14 (https://www.stata.com/stata14/ accessed on 8 June 2023). Data referred to pain (NRS scale) and disability (CONSTANT MURLEY and DASH scales) assessment were treated as ordinal variable using statistical test by ranks Mann–Whitney U-test; data referred to mobility (aROM) and strength (isometric dynamometer) assessment were treated as interval variable using the Student’s t-test. Analysis between groups was conducted for each outcome. Data were also processed for the minimal clinical important difference (MCID) analysis [29,30].

NRS, aROM, Constant Murley, and DASH reference values were obtained from the literature strength; reference values were obtained using the main value of all subjects at each assessment time [31]. 

For missing data, we used the “last observation carried forward” ITT method.

## 3. Results

The study began in October 2017 and was completed in October 2019.

**Figure 1:**
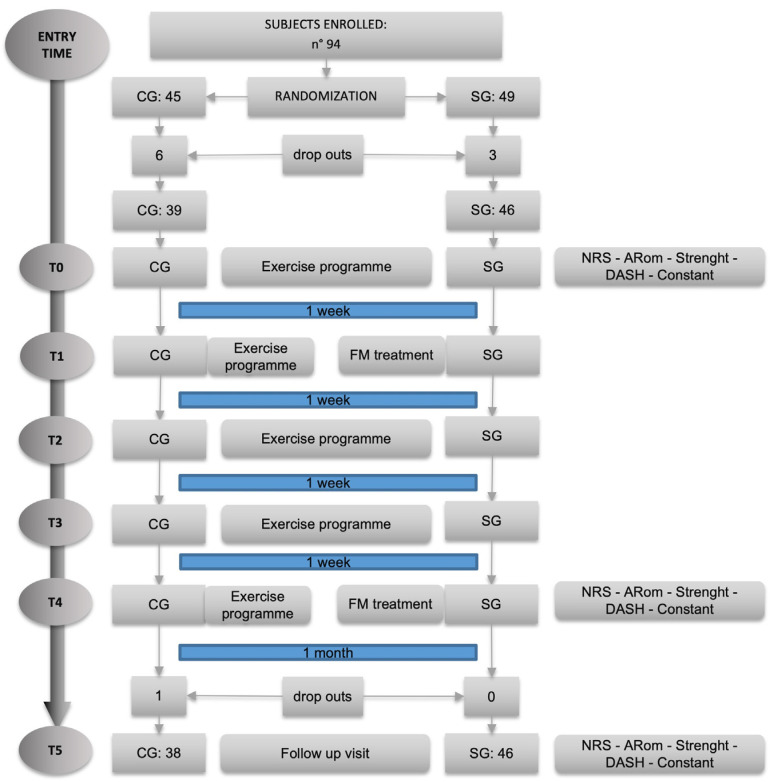
Flow chart of the study: in “Entry Time”, people were enrolled and signed the informed consent and then were randomized into CG and SG; T0 represents the first assessment and the first treatment for both groups; T0–T4 represent the five weekly treatment sessions where T2 and T4 were an FM treatment for SG. As indicated in the right column, assessments were carried out at T0 (before treatment), T4 (after treatment), and T5 (follow-up) visits.

A total of 94 subjects were recruited. In Figure 1 is displayed the flow chart of the study, showing the number of participants recruited and how the assessments were scheduled.

We accepted all the people who volunteered for the study and this allowed us to exceed the number of 35 indicated in the sample size calculation. At the end of the randomization process, SG consisted of 49 subjects and CG of 45 due to the randomization sequence generated by the software; however, nine subjects did not show up for the first evaluation and were therefore excluded from the study. This eventually led to a SG of 46 and a CG of 39 subjects, respectively. 

During the study, only one additional dropout occurred; this was due to work-related injury not related to the activities included in the protocol. No adverse events related to the practices under study were found.

The first assessment carried out at T0 (shown in the Table 1 of the statistical analysis between groups) shows how all the outcome measures in the study define a homogeneity of the two study groups, with the exception of aROM flex which shows a statistically different value between the two groups; this is due to a mean value which is 150.5 (±18.3 SD) for the SG while it is 136.9 (±29.6 SD) for the CG. These data can be seen in Table 2, Table 3 and Table 4. 

For the statistical analysis of the results, we compared the data as follows: at T0 to detect the homogeneity of the two groups at baseline; between T0 and T4 to assess the difference of the various outcomes in the treatment phase of the study; between T0 and T5 to assess the difference of the various outcomes between the start and the end of the study; between T4 and T5 to assess whether the results were maintained in the period when the subjects were not treated.

MCID values are reported with the literature references and data analysis of our study at different session times. Subjects exceeding the MCID values and their percentages within the reference group are reported.

**Figure 2:**
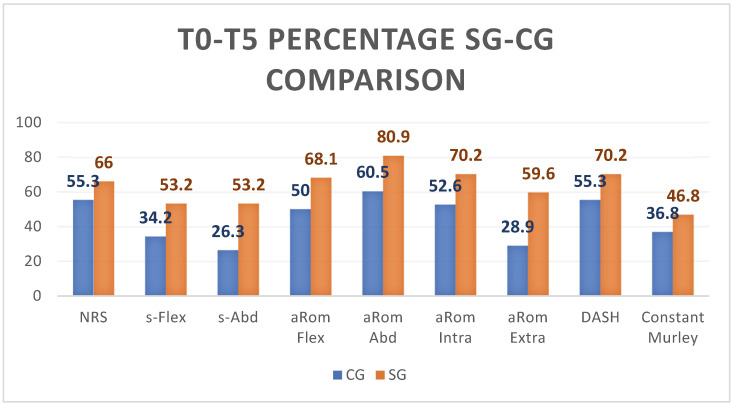
The graph in Figure 2 shows the percentage of the difference between T0–T5 for all the outcomes for CG and SG. SG improved more than CG in every outcome.

In Figure 2, we have summarized the comparison between CG (blue columns) and SG (orange columns) with respect to the difference between the outcome values recorded at T0 and T5: it can be seen that SG improves substantially more than CG for every outcome.

With regard to the study’s primary indicator—pain measured by NRS—the literature reference indicated a 2-point change as clinically significant [30,31,32]. 

The average difference detected between T0 and T4 in the CG and SG shows values above 2, whereas the same comparison between CG and SG between T0 and T5 shows values above 2 only in the SG. Specifically, the percentage of subjects exceeding the MCDI threshold value at T0 and T4 is 57.9% in the CG and 70.3% in the SG; between T0 and T5 it is 55.3% for the CG and 66% for the SG.

With regard to the outcome indicators of s-Flexion and s-Abd, we found no reference standards in the literature to use as MCIDs; in order to try to interpret the findings, we arbitrarily calculated the average value of strength gain over the entire sample of enrolled subjects who completed the study, taking this value as a reference against which to compare the results of each group. 

In the survey at T4, the overall average increase in flexion force was 11.2 Newtons; compared to this value, 51.1% of the SG subjects performed better, compared to 47.4% of the CG subjects. 

At T5, the average overall increase was 13.1 Newtons, a value that was exceeded by 53.2% of the SG subjects and only by 34.2% of the CG subjects. 

As far as force in abduction is concerned, the overall average increase in T4 was 14.9 Newtons, a value exceeded by 57.4% of the subjects in the SG and only 44.7% of the subjects in the CG. At T5, on the other hand, compared to an overall average strength evaluation of the sample of 15.8 Newtons, we saw an improved performance for 53.2% of the subjects in the SG and only 26.3% in the CG. 

A further consideration with regard to the increase in strength can be seen by observing how, while at the end of the period of active reinforcement (T4), the average strength is higher in the CG than in the SG both in flexion (11.8 N compared to 10.6 N) and in abduction (15.8 N vs. 14.1 N); the situation is reversed at follow-up (T5) when, after one month without active reinforcement, the average strength measured is significantly better in the SG than in the CG both in flexion (15.8 N versus 9.9 N) and in abduction (19.0 N versus 11.7 N).

Regarding the variation of aROM flex, the literature indicates 14° as a clinically significant variation [33].

The average measures in both groups were above this value. Specifically, at T4, 72.3% of the SG subjects exceeded the reference threshold, compared with 55.3% of the CG subjects. 

At follow-up, the reference value was exceeded by 68% of SG subjects and only 50% of CG subjects. 

With reference to the variation of the Rom in abduction, the literature reported the MCID value to be 11°.

At the end of the training period, 85.1% of the SG subjects exceeded this increase value, but only 73.3% of the CG subjects exceeded it. At follow-up, the reference value was exceeded by 80.9% of the SG subjects and only 60.5% of the CG subjects [33]. 

The MCDI value for internal and external rotation was indicated in the literature to be equal to 14° [31]. 

With regard to internal rotation at T4, 63.8% of the SG subjects exceeded this value and only 50% of the CG exceeded it. At T5, the percentages were 70.2% in the SG and 52.6% in the CG. 

On the contrary, the change in external rotation was better at T4 for 53.2% of the SG subjects compared to 36.8% of the CG subjects. At follow-up, the percentage of subjects above baseline was 59.6% in the SG and only 28.9% in the CG [33].

In the comparison of the results referring to the Dash scale, the MCDI reported in the literature [34] exceeds the value of 10.83. In the measure at T4, both groups have a higher mean differential value; the same is found in the measurement at follow-up. 

Specifically, in the measure at T4, the percentage variation between T0 and T4 exceeded the reference value in the SG in 57.4% of the subjects, compared to 52.6% in the CG; at follow-up (T5), this variation affected 70.2% of the subjects in the SG, compared to 55.3% of the subjects in the CG. 

The literature taken as a reference for the Constant Murley scale indicated an MCID value of 17 [35].

The average variation values reported between T0 and T4 are not higher than this value for either group; on the other hand, between T0 and T5, the SG exceeds the expected value, unlike the CG. In particular, it is noted that, while at T4 the mean value of the SG is slightly higher than the CG (SG: 12, 25.5% and CG: 13, 34.2%), at T5, values are significantly higher (SG: 22, 46.8% and CG: 14, 36.8%); this trend reversal will be commented on in the discussion. 

Table 1 reports the statistical analysis of the values obtained in this study. 

It can be noted that between T0 and T4 there are no statistically significant differences between the two groups in any of the outcomes found; only the comparison of the ROM value in extrarotation is statistically significant in the comparisons between T0 and T5; more significant are the differences found between T4 and T5, which appear statistically different for the outcomes of strength and again for external rotation.

## 4. Discussion

### 4.1. Results Discussion

The disorders of the shoulder girdle are multiple and complex; among these, pain is a very disabling component that over time can lead to critical dysfunctional situations and to serious difficulties in the activities of daily living.

The importance of physiotherapy in caretaking of subjects with recurrent shoulder pain without instrumental evidence of musculoskeletal injuries is confirmed in this study.

We chose to assess pain, strength, mobility, and functionality as outcomes related to treatment of the shoulder joint. To assess shoulder functionality, we decided to apply the two most widely used scales in literature: DASH and Constant Murley.

In daily practice, soft tissue manipulations, according to FM indications and rules, represent an approach that can offer significant improvement of pain, strength, and functionality, as highlighted in other papers [15,16,36].

For these reasons, we associated an FM treatment to therapeutic exercise in the SG to evaluate if it could be possible to obtain better improvement if compared with physiotherapy exercise alone. With regard to the results of this study, it should be noted the low number of dropouts: the recruitment of subjects in the study, the organization of treatment sessions, the weekly monitoring of the suggested homework, and the little time required to perform the therapeutic exercises (25 minutes a day during four weeks to perform three stretching and three strengthening selective muscle exercises) allowed the subjects to increase their compliance to the study protocol.

The exercises proposed in our protocol are the same that other studies had administered to a sample with similar clinical conditions, resulting in a good improvement in pain and DASH score [7,37].

The intervention protocol for both CG and SG did not show any dropouts or side effects; therefore, we can further state that MF treatment was well tolerated by all SG participants.

In general, all physiotherapists involved in the study gathered verbal comments of good satisfaction from all people in both groups. The recruitment of subjects planned in our study allowed us to analyze a large sample of people with chronic shoulder pain and impairment; their randomization allowed us to identify two statistically homogeneous groups.

The only outcome measure that was statistically different at T0 between the SG and CG (aROM flex) did not invalidate the global data analysis, since comparison of the averages of the two groups at T4 and T5 showed no statistically significant differences. In this study, it was arbitrarily decided that we would use FM through palpatory assessment and treatment of CFs only (see Section 2).

Similarly, it was then agreed that we would administer only two FM treatments three weeks apart, instead of the standard weekly cadence typically indicated in other clinical trials related to FM [15,38]. 

In the last treatment session (T4), all outcome measures were also collected; this resulted in the subjects of the SG being assessed immediately after the treatment of FM. This choice probably affected the values found for a plausible increased sensitization of subjects due to the treatment.

Indeed, in the T0–T4 comparison, not all results showed the expected clinical improvement: almost all of them overcome the MCID threshold; the s-Flex and s-Abd of the SG, the aROM intra- and aROM extrarotation for the CG, and the Constant Murley for both groups did not reach the MCID threshold. 

In the T5 assessments, only the CG showed values lower than the expected MCID with regard to s-Flex, s-Abd, and aROM extra. As discussed above, these results confirm that the FM treatments performed in the SG at T4 influenced the data collected at that time. The effectiveness of the intervention protocol of the study was confirmed by the improvement of the values of all the outcomes for both groups in the comparison between both T4 and T5 to T0.

In the comparison between T4 and T5 it is noted that SG obtained a greater improvement than the CG, confirming the validity of FM treatment (only the aROM flex item made an exception). 

These improvements can be attributed precisely to the deep manual friction implemented by the FM: since we know that many symptomatic conditions such as shoulder pain are based on inflammatory process and that inflammation causes connective tissues alteration, modifying ECM components (and particularly hyaluronic acid behavior increasing its aggregation and size making it adhesive rather than lubricating), FM is able to restore normal tissue gliding due to a deep heating, of at least 40 °C, of the areas where fascia is more stiff and dense; this enables tissues to return to a more fluid and sliding condition, which is associated with a stable reduction of pain and improvement in mobility and functionality [39,40].

Similar results, also stated in other clinical trials referred to the FM [38], are obtained due to a method that assessed and treats not the only painful segment (in our study, the scapular–humeral joint), but considers the anatomical continuity of fascial tissues within the clinical history of each individual. The better sliding of fascial planes would benefit the tensional homeostasis of the myofascial tissues, allowing a stable improvement over time of the results in terms of pain, mobility, and strength.

With regard to functionality, only the DASH scale showed an improvement. This difference could be related to an item recording pain in the DASH scale and not in the Constant Murley which contains items mainly related to mobility. In our study, pain-related scores improved more than mobility-related scores.

Inferential data analysis allowed us to confirm the clinical findings. 

As stated above, we think that no statistically significant difference between T0–T4 was caused by the concurrence at T4 of the last treatment session and the final assessment. The deep fascial treatment often causes temporary soreness, and this most likely affected the assessments at T4.

In addition, the choice to include subjects with a pain value of at least 2 points on the NRS scale conditioned low average values at T0 in both groups (CG = 3.2 ± 2.5 SD; GS = 3 ± 2.3 SD); consequently, it would have been very difficult to obtain a wide reduction of this value and then a statistically significant difference in the between-groups analysis.

The T0–T5 comparison shows statistically significant difference (aROM extra) and others at the limit of the significance parameters (s-Flex and s-Abd) with a better improvement of the SG: this confirms the possible interference of the FM concurrent treatment at T4, and also improvement and maintenance after 4 weeks of the above parameters. 

The T4–T5 between-groups comparison points out the maintenance of the improvements a month after the end of the treatments. The statistical analysis shows better results for SG than the previous analysis, with statistical significant differences in strength (s-Flex and s-Abd), mobility (aROM extra), and functionality (Constant Murley). As shown in Table 4, in the follow-up period, SG continued to increase its results even if people were not treated, on the contrary CG did not maintain the improvements obtained at the end of the treatment period (T4). Confirming what we outlined above, FM enables fascial tissue to restore its fluidity, and with only two FM treatments we can keep this situation stable, confirming similar results of other previous studies [41].

### 4.2. Limits

The limited statistical effectiveness we found in this study could be justified on the following several grounds:

1. We included subjects with mild shoulder pain (at least 2 on the NRS scale); this threshold could not seriously affect mobility and functionality of the shoulder and the upper limb (the mean value of NRS at T0 in SG = 3.3 and in CG = 3.2).
2. Due to the randomization criteria, the number of participants was quite different in the two groups: 47 in SG and 38 in CG. However, the two groups at T0 were statistically homogeneous in every outcome, except for the aROM flex.
3. The probably most influential limit on the results of this study was the decision to treat only the CFs (centers of fusion) as elements of integration and connection between the segmentary motor activations, excluding the CCs (centers of coordination) that are widely used in daily clinical practice.
4. The usual distance between two FM session is one week: we only performed two FM treatments 2 weeks apart. With more and closer FM treatments, we probably could obtain better statistical results.
5. The limited follow-up period (30 days) does not allow us a proper long-term evaluation of the effects of the treatment performed.
6. The aROM and strength assessment at T4 were probably conditioned by the second FM treatment carried out at the same time. The FM technique involves manual friction in the deep fascial tissues, often causing an annoying response and then potentially limiting the following active tests.

### 4.3. Indications for Clinical Practice

Thanks to the protocol implemented and the data collected in this study, we can state the following:

1. A self-administered treatment with simple and easily repeatable exercises protocol, as well as a self-managed treatment diary, are elements that facilitate adherence to treatment by participants.
2. The treatments in the study (active exercises, stretching exercises, and FM treatment) did not show any side effects and were well appreciated by all the subjects.
3. Using both CCs and CFs in FM treatments represents a more comprehensive treatment choice with potentially better results.
4. Using a weekly schedule for FM treatment and planning more than two FM treatments, as indicated in most clinical trials, could further improve clinical outcomes.

FM treatment may adversely affect the assessments scores if collected immediately after treatment. A more truthful and reliable evaluation should be performed at least one week after the FM treatment.

## 5. Conclusions

A physiotherapy approach is effective for the treatment of recurrent shoulder pain. The treatment includes stretching exercises and a selective muscle reinforcement program, which are also self-administered well by subjects.

The use of a self-managed diary and the delivery of an audio–visual support (a DVD with video description of the exercises) allowed all the subjects to completely adhere to the protocol of the study.

Two FM sessions enabled subjects in the SG to better improve in the short term (30 days) their active mobility and muscle strength without showing any side effects at all.

From a clinical point of view, considering MCID values, our protocol of intervention demonstrates that a specific physiotherapy program reduces pain and allows an improvement in both active mobility and activities of daily life. In the last follow-up, subjects in SG showed better results than CG, showing that FM treatment should be performed for nonspecific shoulder pain. 

Moreover, as stated above in the Section 4.2, a more faithful application of the FM technique, as indicated by the authors and applied in other studies, could further improve the outcomes in subjects with nonspecific shoulder pain.

## Figures and Tables

**Table 1:** Between-groups statistical analysis.

Between Group Analysis	T0		T0–T4		T0–T5		T4–T5	
	*p*-Value	95% C.I.	*p*-Value	95% C.I.	*p*-Value	95% C.I.	*p*-Value	95% C.I.
NRS	0.653	(-)	0.407	(-)	0.107	(-)	0.115	(-)
s-Flex	0.116	−17.921 1.996	0.711	−5.356 7.821	0.057	−11.982 0.174	0.001	−11.360 −2.914
s-Abd	0.089	−20.775 0.499	0.656	−5.896 9.313	0.052	−14.657 0.0759	0.001	−14.274 −3.725
aROM Flex	0.011	−23.971 −3.176	0.879	−8.727 10.181	0.628	−8.698 14.331	0.444	−3.3197 0.498
aROM Abd	0.235	−23.004 0.728	0.546	−13.980 7.451	0.210	−20.3694 0.537	0.170	−11.330 2.0267
aROM Intra	0.255	−9.235 2.487	0.223	−9.881 2.336	0.064	−14.087 0.415	0.298	−8.878 2.751
aROM Extra	0.276	−2.998 10.350	0.075	−13.251 0.646	0.001	−21.033 −5.287	0.024	−12.804 −0.911
DASH	0.972	(-)	0.333	(-)	0.1398	(-)	0.580	(-)
Constant Murley	0.100	(-)	0.714	(-)	0.269	(-)	0.017	(-)

Table 1 reports statistical analysis. In the 1st column are listed all the outcomes; 2nd and 3rd columns report T0 analysis to show the homogeneity of groups; 4th and 5th columns report the T0–T4 analysis; 6th and 7th columns report the T0–T5 analysis; 8th and 9th columns report the T4–T5 analysis. For each analysis, *p*-value and 95% confidence interval (C.I.), if indicated, are reported.

**Table 2:** T0–T4 analysis.

T0–T4 Analysis		NRS	s-Flex	s-Abd	aROM Flex	aROM Abd	aROM Intra	aROM Extra	DASH	Constant Murley
Mean	CG	2.2	11.8	15.8	22.7	29.4	12.6	10.0	13.7	13.6
Mean	SG	2.7	10.6	14.1	22.0	32.7	16.4	16.3	17.6	12.4
**MCID**		**2**	**11.2**	**14.9**	**14**	**11**	**14**	**14**	**10.83**	**17**
MCID (n)	CG	22	18	17	21	28	19	14	20	13
MCID (n)	SG	33	24	27	34	40	30	25	27	12
MCID (%)	CG	57.9	47.4	44.7	55.3	73.7	50.0	36.8	52.6	34.2
MCID (%)	SG	70.2	51.1	57.4	72.3	85.1	63.8	53.2	57.4	25.5

Table 2 reports T0–T4 differences for each group in every outcomes. In the left column 1st row: the mean differences of the CG; 2nd: the mean differences of the SG; 3rd: the reference MCID values; 4th: the numbers (n) of subjects of the CG which exceeded the MCID reference value; 5th: the numbers (n) of subjects of the SG which exceeded the MCID reference value; 6th: the percentage (%) of subjects of the CG which exceeded the MCID reference value; 7th: the percentage (%) of subjects of the SG which exceeded the MCID reference value.

**Table 3:** T0–T5 analysis.

T0-T5 Analysis		NRS	s-Flex	s-Abd	aROM Flex	aROM Abd	aROM Intra	aROM Extra	DASH	Constant Murley
Mean	CG	1.7	9.9	11.7	22.3	26.1	14.1	5.1	13.7	12.3
Mean	SG	2.6	15.8	19.0	19.4	34.0	20.9	18.2	19.3	13.9
**MCID**		**2**	**13.1**	**15.8**	**14**	**11**	**14**	**14**	**10.83**	**17**
MCID (n)	CG	21	13	10	19	23	20	11	21	14
MCID (n)	SG	31	25	25	32	38	33	28	33	22
MCID (%)	CG	55.3	34.2	26.3	50.0	60.5	52.6	28.9	55.3	36.8
MCID (%)	SG	66.0	53.2	53.2	68.1	80.9	70.2	59.6	70.2	46.8

Table 3 reports T0–T5 differences for each group in every outcomes. In the left column 1st row: the mean differences of the CG; 2nd: the mean differences of the SG; 3rd: the reference MCID values; 4th: the numbers (n) of subjects of the CG which exceeded the MCID reference value; 5th: the numbers (n) of subjects of the SG which exceeded the MCID reference value; 6th: the percentage (%) of subjects of the CG which exceeded the MCID reference value; 7th: the percentage (%) of subjects of the SG which exceeded the MCID reference value.

**Table 4:** T4–T5 analysis.

T4-T5 Analysis		NRS	s-Flex	s-Abd	aROM Flex	aROM Abd	aROM Intra	aROM Extra	DASH	Constant Murley
Mean	CG	−0.5	−2.0	−4.1	−0.4	−3.3	1.4	−4.9	−0.1	−1.3
Mean	SG	−0.1	5.2	4.9	−2.5	1.4	4.5	2.0	1.7	1.6
**MCID**		**2**	**2.0**	**0.9**	**14**	**11**	**14**	**14**	**10.83**	**17**
MCID (n)	CG	2	14	15	6	7	7	4	5	2
MCID (n)	SG	3	28	31	2	9	15	10	9	1
MCID (%)	CG	5.3	36.8	39.5	15.8	18.4	18.4	10.5	13.2	5.3
MCID (%)	SG	6.4	59.6	66.0	4.3	19.1	31.9	21.3	19.1	2.1

Table 4 reports T4–T5 differences for each group in every outcomes. In the left column 1st row: the mean differences of the CG; 2nd: the mean differences of the SG; 3rd: the reference MCID values; 4th: the numbers (n) of subjects of the CG which exceeded the MCID reference value; 5th: the numbers (n) of subjects of the SG which exceeded the MCID reference value; 6th: the percentage (%) of subjects of the CG which exceeded the MCID reference value; 7th: the percentage (%) of subjects of the SG which exceeded the MCID reference value.

## Data Availability

The data presented in this study are available on request from the corresponding author. The data are not publicly available due to privacy reasons.

## Appendix A. The Daily Exercise Program

**Table A1:** Daily exercise program.

Stretching Exercises		
Exercise 1	Exercise 2	Exercise 3
Upper trapezius stretch	Pectoralis minor stretch	Posterior shoulder stretch

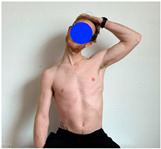
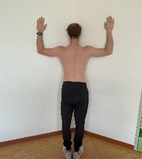
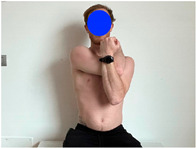
		
**Strengthening Exercises**		
Exercise 1	Exercise 2	Exercise 3
Shoulder external rotation	Shoulder extension targeting lower trapezius strengthening	Shoulder protraction targeting serratus anterior strengthening

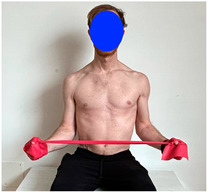
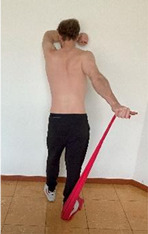
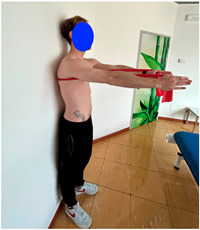
		

Table A1 shows the daily exercise program for every participants. These exercises were supervised, arranged and adapted for each individual during the exercise sessions (5 for CG, 3 for SG).
